# Screening and analysis of the risk of dysphonia based on general and specific screening protocols in teachers with and without voice disorders

**DOI:** 10.1590/2317-1782/e20250175en

**Published:** 2026-03-30

**Authors:** Eric Rodrigues Dias, Renata da Silva Gonçalves, Marcia Simões-Zenari, Katia Nemr

**Affiliations:** 1 Departamento de Fisioterapia, Fonoaudiologia e Terapia Ocupacional, Faculdade de Medicina, Universidade de São Paulo – USP – São Paulo (SP), Brasil.

**Keywords:** Voice, Teachers, Risk Factors, Dysphonia, Speech Therapy

## Abstract

**Purpose:**

To assess the risk of dysphonia in teachers with and without voice disorders, compare the scores of the screening protocols, and correlate the risks with the overall severity of voice disorder.

**Methods:**

Teachers from different education levels and institutions, of both sexes, aged over 18 years, participated in the study. The study applied the General Dysphonia Risk Screening Protocol (PRRD-G) and the Specific Dysphonia Risk Screening Protocol for Teachers (PRRD-Pro) and recorded and analyzed voice samples. Participants were divided into two groups, with and without voice disorders, based on the mean overall severity in the Consensus Auditory-Perceptual Evaluation of Voice (CAPE-V). Descriptive analyses and comparisons of the protocol data between the groups were performed.

**Results:**

The groups were homogeneous regarding sex and age. There was no statistically significant difference between the groups in PRRD-G, PRRD-Pro, and total scores. The smoking subscore was worse in the group with voice disorders, and hydration was worse in the group without disorders. There was no correlation between the overall severity and the protocol scores.

**Conclusion:**

The mean PRRD-G scores were above the cutoff in both groups, indicating a high risk of dysphonia, even in the absence of voice disorders. Smoking and hydration were relevant in differentiating between teachers with and without voice disorders. The study is planned to continue with a larger sample size, laryngological examination, observation of voice and communication in the classroom, vocal improvement workshops, and speech therapy.

## INTRODUCTION

The voice is fundamental in teaching for transmitting knowledge to students; hence, it requires special care. Teachers, who use the voice as the main tool of their trade, are occupational voice users, being therefore more susceptible to occupational voice disorders (OVD)^(1)^. Individual anatomical and physiological characteristics, workload, physical environment conditions, and the level of education they teach can interfere with the likelihood of developing vocal changes. Thus, they may have to be absent from work and suffer impacts on their work activity and quality of life.

The common vocal signs and symptoms reported by teachers in this occupational pathology include vocal fatigue, hoarseness, vocal tract discomfort, tiredness during and after voice use, worsened symptoms with prolonged voice use, excessive effort to speak, head and neck tension and pain, decreased vocal projection, episodes of aphonia, voice breaks and failures, weak voice, throat clearing, and so forth^(2-5)^.

Voice disorder screening instruments can identify and monitor all these manifestations; the more present they are, the greater the risk of dysphonia. The General Dysphonia Risk Screening Protocol (PRRD-G), as a proposal that expands this investigation, has proven sensitive in differentiating people with and without vocal complaints or dysphonia, and verifies the risk of dysphonia in different groups due to its applicability across a wide age range, regardless of gender, sex, and voice use. This protocol encompasses subjective and objective voice-related topics to screen for signs and symptoms, disease history, hydration, family voice changes, and so on^(6)^.

It can be associated with specific dysphonia risk screening protocols, according to the professional group, to complement and analyze the risks more robustly, as each profession has specific demands, needs, and risks in vocal use at work^(7)^. A study applied the PRRD-G and a specific dysphonia screening pilot protocol for teachers (Dysphonia Risk Screening Protocol - Complementary Occupational Voice Users – Teachers [PRRD-Pro]) to preschool and elementary school teachers and verified its sensitivity to differentiate the participants’ particularities^(6,7)^.

These tools enable screening, guidance, prevention of voice disorders, collaboration with therapeutic practice, and improved teachers' self-perception of risks in personal and work environments. Thus, attention to higher subscores can motivate changes in habits and behaviors that promote greater vocal well-being and reduce the conditions that generate or aggravate voice disorders in this population.

Although the teacher's voice is widely studied, few research projects have longitudinally monitored their risk of dysphonia. Thus, this research comprises stages such as risk assessment, classroom observation, proposals for workshops and speech therapy, and longitudinal monitoring of vocal risks. The objective, in this first stage, was to investigate the general and specific risk of dysphonia in teachers with and without voice disorders, to correlate the risk of dysphonia with the overall degree of vocal deviation, and to present the final proposal of the PRRD-Pro.

## METHODS

Cross-sectional research approved by the institution's Research Ethics Committee (CAAE No. 87344318.0.0000.0065). All participants signed an informed consent form.

The proposal was presented to two educational institutions, one public and one private, both in the municipality of São Paulo, and teachers linked to them, aged 18 years or older, were invited to participate, regardless of the level of education and gender. Those who did not complete the study stages were excluded.

Considering the sample size calculation, 79 teachers participated, divided, according to voice disorders, into a control group (CG): n = 34, being 27 women and seven men, with a mean age of 39.56 years (SD = 7.64) and a mean overall severity of vocal deviation of 26.56 (SD = 8.53); and an experimental group (EG): n = 45, with 35 women and 10 men, mean age of 42.42 years (SD = 10.44) and a mean overall severity of 45.47 (SD = 9.53). The groups were similarly distributed regarding age and gender, with homogeneity in these variables (p = 0.157).

Data were collected at the participants' workplaces, in a controlled and quiet environment, at two time points (previous study^(7)^ and current study). In the previous study^(7)^, teachers from a private school participated. A new collection, in a public school, was added to this previous sample, thus composing the sample for the current study. Participants completed the PRRD-G^(6)^ and PRRD-Pro, made available in printed form or online (via Google Forms). The research team was present in both contexts to answer any questions.

Each protocol allows the calculation of partial subscores and the final score. The subscores are calculated from the sum of the scores assigned to each item of the thematic blocks; the items can range from 0 (no risk) to 3 (highest risk). The subscores are added together in the final score, which can range from 0 to 131 (PRRD-G) and from 0 to 44 (PRRD-Pro). The final PRRD-G and PRRD-Pro scores were added together for analysis of the total score.

The PRRD-G cutoff for high risk is defined in its validation study, being 29.25 for adult women, 22.75 for adult men, and 27.10 for older people of both sexes^(6)^. The present analysis considered these scores.

The initially proposed PRRD-Pro consisted of 13 questions, 12 of which were aimed at all teachers, regardless of gender, and involved daily voice use, vocal rest, warm-up and cool-down, type of teacher activity (general/lead teacher, specialist, or coordinator), average number of students per class, teaching level, school grade, teaching time, physical conditions of the workplace, use of vocal amplification resources, absence from work due to dysphonia, smoking, alcohol consumption, use of illicit drugs, aspects related to the use of dental prostheses, and a block of specific questions on women's health.

Voice samples were recorded after completing the protocols. The initial collection used the Zoom H4 recorder, positioned perpendicularly 30 centimeters from the participant's mouth^(7)^. The current collection used the Shure^®^ MV88+ microphone, positioned 10 centimeters from the mouth^(8)^ and connected to an Apple iPad^®^. The study used the tasks proposed in the Consensus Auditory-Perceptual Evaluation of Voice (CAPE-V)^(9)^, considering the overall severity of vocal deviation.

Two speech-language-hearing pathologists with more than 5 years of experience in this type of analysis performed the auditory-perceptual evaluation of the entire sample in this study. Random and blind repetition of the voices of 20 individuals from the sample was added to the sample for reliability analysis. A two-way model with fixed effects based on the absolute agreement of single measures was used in the calculation of the intraclass correlation coefficient (ICC), and the interrater reliability was moderate (0.520). The simple average between judges was calculated from each one’s analysis, generating the final overall severity of vocal deviation, and extracting the mean, median, standard deviation, minimum, and maximum values. The group that scored between 0 and 35.5 (CG) was considered normal, and those above 35.5 (EG) were considered abnormal^(9)^.

Descriptive and inferential statistical analyses considered the following variables: sex; age; overall severity of vocal deviation; PRRD-G score; PRRD-Pro score; total score (sum of PRRD-G and PRRD-Pro scores); PRRD-G subscores: visual analog scale (VAS), signs and symptoms (SS), voice use outside of work (VO), diet (DI), hydration (HY), sleep (SL), history of illnesses (HI), previous voice disorders (PD), medications (MD), and contact with smokers (CS); and PRRD-Pro subscores: professional experience (PE), teaching aspects (TA), workplace physical conditions (WP), women's health (WH), smoking (SM), and dental prosthesis (DP).

Student’s t-test was used to compare EG with CG regarding PRRD-G, PRRD-Pro, and total scores. Student’s t-test with Welch correction factor and Fisher's exact test were used to compare them regarding PRRD-G and PRRD-Pro subscores. Pearson's correlation test was used for correlation analysis between protocol scores (PRRD-G, PRRD-Pro, and total score) and the overall severity of vocal deviation.

Statistical analyses used the 5% significance level (p ≤ 0.05) and SPSS, version 27.0 (IBM Corp., Armonk, NY, USA). The results were also interpreted based on the effect size.

## RESULTS

The mean PRRD-G, PRRD-Pro, and total scores were close between the groups, with similar risk, regardless of the presence or absence of voice disorders. The mean PRRD-G scores were above the cutoff for high risk of dysphonia in both groups. The comparison of scores between the groups found no statistically significant difference or relevant effect size, as shown in Table 1.

**Table 1:** Characterization and comparison of the groups regarding the PRRD-G, PRRD-Pro, and total scores

**Scores**	**CG**		**EG**		**Diff.**	**95% CI**	**df**	**p**	**ES**
	**Mean (SD)**	**Median (Min–Max)**	**Mean (SD)**	**Median (Min–Max)**					
PRRD-G	40.89	40.90	42.94	40.00	-2.04	-8.92–4.42	77	0.532	0.072
	(14.14)	(10–66)	(15.60)	(16–88.7)					
PRRD-Pro	21.53	22.00	21.49	22.00	0.41	-2.03–2.19	77	0.971	0.004
	(4.27)	(12–30)	(5.32)	(12–34)					
Total	62.13	66.00	64.42	61.00	-2.30	-9.22–4.96	77	0.553	0.069
	(15.96)	(34–90)	(17.95)	(31–118.70)					

**Caption:** SD: standard deviation; Min: Minimum; Max: Maximum; Diff.: difference between means; CI: confidence interval; df: degrees of freedom; ES: effect size

The comparison of the groups regarding PRRD-G subscores found a statistically significant difference in hydration (HY) – i.e., the scores of teachers without voice disorders were higher than those of the group with disorders, indicating a higher risk in the hydration subscore in CG. The comparison of the groups regarding PRRD-Pro subscores found a statistically significant difference in smoking (SM), with higher scores for teachers with voice disorders, indicating a higher risk in the smoking subscore in EG, as detailed in Table 2.

**Table 2:** Characterization and comparison of the groups regarding the PRRD-G and PRRD-Pro subscores

	**CG**		**EG**		**Diff.**	**95% CI**	**df**	**p**	**ES**
	**Mean (SD)**	**Median (Min–Max)**	**Mean (SD)**	**Median (Min–Max)**					
**PRRD-G**									
VAS	3.69	4.00	4.69	5.00	-1.01	-2.12–0.05	77	0.073	0.209^†^
	(2.23)	(0–8)	(2.53)	(0–10)					
SS	23.35	21.50	24.73	22.00	-1.38	-6.21–3.77	77	0.611	0.017
	(11.12)	(2–46)	(11.81)	(6–59)					
VO	2.65	3.00	2.71	3.00	-0.06	-0.58–0.48	77	0.803	0.027
	(1.28)	(0–5)	(1.06)	(0–5)					
DI	2.29	2.00	1.98	2.00	0.32	-0.21–0.82	77	0.269	0.126^†^
	(1.24)	(0–5)	(1.22)	(0–4)					
HY	1.50	2.00	0.96	0.00	0.54	-0.06–1.11	77	0.047*	0.224^†^
	(1.19)	(0–3)	(1.22)	(0–3)					
SL	1.26	1.00	1.02	1.00	0.24	-0.30–0.79	63.7	0.374	0.111^†^
	(1.29)	(0–3)	(1.08)	(0–3)					
HI	1.56	1.00	1.29	1.00	0.27	-0.48–0.98	77	0.462	0.083
	(1.64)	(0–5)	(1.66)	(0–6)					
PD	1.56	2.00	1.60	2.00	-0.04	-0.35–0.30	77	0.803	0.030
	(0.70)	(0-2)	(0.72)	(0-2)					
MD	0.29	0.00	0.56	0.00	-0.26	-0.52 - 0.01	76.1	0.070	0.207^†^
	(0.58)	(0-2)	(0.69)	(0-2)					
CS	0.26	0.00	0.58	0.00	-0.31	-0.67–0.04	75.2	0.088	0.199†
	(0.621)	(0-3)	(0.97)	(0-3)					
**PRRD-Pro**									
PE	2.79	3.00	3.02	3.00	-0.23	-0.82–0.39	77	0.466	0.083
	(1.41)	(1–7)	(1.36)	(1–7)					
TA	9.76	10.00	8.98	9.00	0.79	-0.20–1.73	77	0.116	0.176^†^
	(2.08)	(6–12)	(2.39)	(3–12)					
WP	6.85	7.00	7.07	7.00	-0.21	-1.33–0.82	77	0.690	0.046
	(2.11)	(3–11)	(2.80)	(2–14)					
WH	0.82	1.00	0.78	1.00	0.05	-0.25–0.34	77	0.759	0.034
	(0.76)	(0–3)	(0.56)	(0–2)					
SM	0.12	0.00	0.51	0.00	-0.39	-0.78–0.00	67.2	0.041*	0.243†
	(0.54)	(0-3)	(1.10)	(0-3)					
DP	0.03	0.00	0.04	0.00	-0.02	-0.10–0.07	77	0.743	0.039
	(0.17)	(0-2)	(0.21)	(0-2)					

Student’s t-test

* statistically significant;

† small effect

**Caption:** SD: standard deviation; Min: minimum; Max: maximum; Diff.: difference between means; CI: confidence interval; df: degrees of freedom; ES: effect size; VAS: visual analog scale; SS: signs and symptoms; VO: voice use outside of work; DI: diet; HY: hydration; SL: sleep; HI: history of illnesses; PD: previous voice disorders; MD: medications CS: contact with smokers; PE: professional experience; TA: teaching aspects; WP: workplace physical conditions; WH: women's health; SM: smoking; DP: dental prosthesis

The mean overall severity score was not statistically significantly correlated with the PRRD-G scores (p = 0.315), PRRD-Pro scores (p = 0.962), or total scores (p = 0.262), and the effect size was small.

## DISCUSSION

This study investigated the risk of dysphonia in teachers, compared the risk between teachers with and without voice disorders, and analyzed the correlation between risk and overall severity of vocal deviation.

The group with voice disorders comprised teachers with mild to moderate deviations, similar to another study with 60 teachers with dysphonia^(10)^. Mild voice disorders often go unnoticed by these professionals, who end up not seeking speech-language-hearing therapy^(10)^.

There was a similar distribution between the groups regarding age and proportion between the sexes. However, the predominance of females in both groups reflects the composition of the teaching population, and the few male participants may influence generalizations regarding the influence of sex on the findings. This reinforces the importance of considering factors related to working conditions and occupational voice use, beyond biological variables, as paramount for analyzing the risk for OVD^(1,10)^.

Regarding the protocol data, PRRD-G and PRRD-Pro scores were similar in both groups of teachers, with high scores, indicating a high risk of dysphonia. This finding suggests that the risk for dysphonia is similar for these professionals, in the general and specific context of voice use, regardless of the presence of dysphonia, work dynamics, and institution, as observed previously^(7)^. These data are reinforced by the lack of correlation between the risk of dysphonia and the severity of vocal deviation.

On the other hand, two subscores differentiated the groups: smoking and hydration, although with a small effect size.

Regarding hydration, teachers from both groups reported ingesting amounts of water below the minimum recommended, being only slightly higher in the group with voice disorders, perhaps in an attempt to alleviate greater discomfort during voice use. Such data should be further explored in the future, and the relationship between the lack of adequate hydration and the presence of symptoms such as dry throat, throat clearing, and vocal fatigue should be reinforced among teachers^(3)^. Systemic hydration, considered ideal, is very accessible for teachers in the classroom^(11)^, and its positive effects on vocal aspects are known, including when water intake occurs after vocal loading, with participants reporting less phonatory effort after hydrating^(12)^.

Smoking had a low occurrence in both groups, slightly higher in teachers with voice disorders. The harmful effects of smoking on general health, especially on the larynx and voice, are a consensus in scientific literature. High concentrations of harmful substances irritate, inflame, and dry the mucous membranes, changing the acoustic and auditory-perceptual properties of the voice^(13,14)^. A study with 357 university professors recorded worse scores in quality-of-life protocols and voice handicap in smokers than in non-smokers, limiting voice use, including during professional practice. They also had increased daily vocal complaints and other discomforts in the respiratory system^(14)^.

Smoking is directly related to voice disorders. The literature has consolidated its harmful effects on the voice, with Reinke's edema being one of the most frequent laryngeal conditions in smokers, with a significant impact on the voice^(15)^. Aspects observed by the researchers during data collection were listed at the end of this preliminary PRRD-Pro investigation and modified in the final version (Appendix A). Thus, the subitems "remote learning," "mental health," and "orthodontic appliances/dental aligners" were added, the subitem "smoking" was detailed, and the scoring of the subitems "drug use" and "dental prosthesis" was modified.

This study contributes to the scientific production on the teacher's voice. The findings reinforce the need for further analysis of dysphonia risk indicators, especially those intrinsic to teaching. The association between PRRD-G and PRRD-Pro, together with voice assessment, provides quantitative and qualitative data that can guide future speech-language-hearing actions. The findings regarding hydration and smoking highlight the need to incorporate these aspects as a priority in prevention and intervention strategies.

Finally, further studies should increase the sample to confirm the findings and explore other ways of dividing the groups, given that the average severity of voice disorder in the present sample was mild. Participants are being observed during classes and are also gradually referred for laryngological examination. All resulting data will be considered in the complete study.

## CONCLUSION

The mean PRRD-G scores were above the cutoff for both women and men, representing a high risk of dysphonia in both groups of teachers, with and without voice disorders.

There was no difference between the groups regarding the final mean scores (PRRD-G, PRRD-Pro, and total scores), and the risk of dysphonia was not correlated with the overall severity of vocal deviation, demonstrating that the high risk of dysphonia occurred independently of the presence of voice disorders. Only the hydration and smoking subscores had small differences between the groups.

It was possible to adapt and finalize the dysphonia screening protocol specific to teachers.

## Appendix A Dysphonia Risk Screening Protocol - Complementary Occupational Voice Users – Teachers

Dysphonia Risk Screening Protocol - Complementary

Occupational Voice Users – Teachers

PRRD-Pro

**General instructions:** the scores that go into the calculation of each subscore (PE, WU, TA, WP, AW, SM, AL, DR, DP, OL, WH) and the guidelines regarding these scores are highlighted in gray. If the teacher works at more than one institution, a PRRD-Pro form must be completed for each school.

NAME: ___________________________________________ Date: _____/_____/_________

1. Time working as a teacher: __________________________________________________

**PE:**__ 2. Average time spent using the voice **per day**:

2.1. weekdays: works for __________ hours and uses the voice for __________ hours

(consider 0 = up to 2 hours of use/day, 1 = 2:01 to 5:00, 2 = 5:01 to 8:00, 3 = more than 8 hours of use/day)

2.2. weekend: works for _________ hours and uses the voice for __________ hours

(*consider 0 = up to 2 hours of use/day, 1 = 2:01 to 5:00, 2 = 5:01 to 8:00, 3 = more than 8 hours of use/day*)

2.3. Does he/she take breaks to allow the voice to rest? 1. ( ) no 0. ( ) yes; **if so**, describe the average duration of pauses and how often they occur:

**WU:**__ 3. Voice warm-up/cool-down:

3.1. Warm-up ( ) no ( ) yes

3.2. Cool-down ( ) no ( ) yes

**If so** (both or at least one of the two), describe (which situations, how often, and what procedures): _______________________________________________________________

__________________________________________________________________________

consider 0 = yes, warm-up and cool-down; 2 = only warm-up or only cool-down; 3 = doesn’t warm up or cool down)

**TA:**__ 4. Describe:

4.1. Are you a teacher of a specific subject (specialist) or a general/lead/generalist teacher?

( 1 )* specialist; which subject: ( )** ________________________________________

( 3 )* general/generalist/lead

( 0 )* others; describe: _____________________________________________________

*(*consider 0 = other; 1 = specialist teacher; 3 = general/lead teacher*)

**(c*onsider 1 = arts, languages, etc.; 2 = physical education*)

4.2. Average number of students per class/day: ( ) _________________________________

__________________________________________________________________________

(consider 1 = Up to 15 students; 2 = From 15 to 30 students; 3 = More than 30 students)

4.3. Education level and grade level currently teaching: ( ) ___________________________

__________________________________________________________________________

(c*onsider 0 = higher education; 1 = high school; 2 = elementary school; 3 = preschool*)

4.4. How long have you been teaching this level/grade?: ( ) ___________________________

__________________________________________________________________________

(*consider 0 = From 6 months to 2 years; 2 = From 2 to 4 years; 3 = More than 4 years*)

**WP:**__ 5. Environmental conditions at work:

( 2 ) noise (internal/external)

( 2 ) dust

( 2 ) air conditioning

( 2 ) chalk dust

( 1 ) open space

( 1 ) very large place

( 1 ) very hot environment

( 1 ) very cold environment

( 2 ) irritating chemicals

( 1 ) other: _________________________

(*consider 0 = none checked; 1 = if open space, very large space, very hot environment, very cold environment, or others are checked (1 point for each); 2 = if internal/external noise, dust, air conditioning, chalk dust, or irritating chemicals are checked (2 points for each); calculate the WP subscore by summing the scores of all those checked above*)

6. Do you use a microphone or other vocal amplification device?

( ) no ( ) yes; describe (daily usage time, model, whether training was provided): ___________

____________________________________________________________________________________________________________________________________________________

6.1. **If not**, do you find it necessary to use it? ( ) no ( ) yes; if so, describe: _______________

____________________________________________________________________________________________________________________________________________________

**AW:**__ 7. Have you ever been absent from work because of your voice? ( 0 ) no ( 2 ) yes; **if so**, describe:

__________________________________________________________________

**SM:**__ 8. Regarding traditional cigarette smoking:

( 0 ) never smoked

( 0 ) former smoker more than 10 years ago; how many years ago did you quit? ____________; average number of cigarettes smoked: __________

( 1 ) former smoker less than 10 years ago; how many years ago did you quit? _____________; average number of cigarettes smoked: ___________

( 3 ) smoker; how many years have you been smoking _______________________________; average number of cigarettes/day _____________

8.1. Besides traditional tobacco, do you have any other smoking habits (electronic cigarettes, cigars, pipes, hookahs, hand-rolled cigarettes, etc.)? ( ) no ( ) yes

. pipe: ( 1 ) seldom; ( 2 ) sometimes; ( 3 ) always

. cigar: ( 1 ) seldom; ( 2 ) sometimes; ( 3 ) always

. hand-rolled cigarette: ( 1 ) seldom; ( 2 ) sometimes; ( 3 ) always

. cigarillo: ( 1 ) seldom; ( 2 ) sometimes; ( 3 ) always

. electronic cigarette: ( 1 ) seldom; ( 2 ) sometimes; ( 3 ) always

. hookah: ( 1 ) seldom; ( 2 ) sometimes; ( 3 ) always

. other: ___________ ( 1 ) seldom; ( 2 ) sometimes; ( 3 ) always

(*To calculate the SM subscore, sum the scores of all those marked under 8 and 8.1.*)

**AL:**__ 9. Do you drink alcohol?

( 0 ) no ( 1 ) yes; **If so**, describe: type of drink, quantity, and frequency of consumption: __________________________________________________________________________

**DR:**__ 10. Do you use or have you used drugs?

( 0 ) no ( 2 ) yes; **If so**, describe: type, quantity, and frequency of use: __________________

__________________________________________________________________________

**DP:**__ 11. Do you wear dental prostheses (such as dentures or a bridge; do not consider crowns or dental implants)?

( ) no ( ) yes

**If not**: Do you have any indication for use? ( ) no ( ) yes

**If so**, describe the type and how long you have been using it:___________________________ __________________________________________________________________________

**If so**, do you have any complaints regarding the use of this prosthesis? ( ) no ( ) yes; describe: _________________________________________________________________________

11.1. Do you wear orthodontic braces (whether fixed, removable, or aligners like Invisalign)?

( ) no ( ) yes

**If not**: Do you have any indication for use? ( ) no ( ) yes

**If so**, describe the type and how long you have been using it:___________________________ __________________________________________________________________________

**If so**, do you have any complaints regarding the use of this prosthesis? ( ) no ( ) yes; describe:

__________________________________________________________________________ *(consider, in both 11 and 11.1: 0 = does not use and has no indication for use; 1 = uses and has a complaint regarding its use; 2 = has an indication for use, but does not use; to calculate the DP subscore, add the scores from 11 and 11.1.*)

**OL:**__ 12. Do you teach online classes?

( ) no ( ) yes; describe (frequency, location, subject, average duration of each class): __________________________________________________________________________

__________________________________________________________________________

**If so**, do you experience any physical discomfort (back pain, shoulder pain, neck pain, etc.) during or after online classes? ( 0 ) no ( 1 ) yes; describe: _____________________________

__________________________________________________________________________

**If so**, Do you experience any vocal discomfort? ( 0 ) no ( 1 ) yes; describe: ________________

__________________________________________________________________________

(to calculate the OL subscore, sum the scores marked in gray)

**WH:**__ 13. **For women only**:

13.1. Do you have symptoms of premenstrual tension? ( 0 ) no ( 1 ) yes; if so, describe:

__________________________________________________________________________

__________________________________________________________________________

13.2. Are you pregnant? ( 0 ) no ( 1 ) yes; if so, gestation week: _______________________

__________________________________________________________________________

13.3. Are you currently experiencing menopause or post-menopause? ( 0 ) no ( 1 ) yes; if so, for how long? _______________________________________________________________

__________________________________________________________________________

13.4. Do you have hormonal problems? ( 0 ) no ( 1 ) yes; if so, describe: ________________

____________________________________________________________________________________________________________________________________________________

13.5. Do you use birth control? ( 0 ) no ( 1 ) yes; if so, describe the type and duration of use: __________________________________________________________________________

14. Would you like to add any information? _______________________________________

____________________________________________________________________________________________________________________________________________________

**FINAL PRRD-**___

**ProSCORE**____

_____________________
