# Supplementary material for: Screening and analysis of the risk of dysphonia based on general and specific screening protocols in teachers with and without voice disorders
Source: Codas. 2026 Mar 30;38(2):e20250175. doi: 10.1590/2317-1782/e20250175en (PMC13075848; doi:10.1590/2317-1782/e20250175en)
Supplement: Translation

# Translation: Rastreio e análise do risco de disfonia a partir de protocolos de rastreio geral e específico em professores com e sem alteração vocal

**DOI:** 10.1590/2317-1782/e20250175pt

**Authors:** Eric Rodrigues Dias, Renata da Silva Gonçalves, Marcia Simões-Zenari, Katia Nemr

**Affiliations:** 1 Departamento de Fisioterapia, Fonoaudiologia e Terapia Ocupacional, Faculdade de Medicina, Universidade de São Paulo – USP – São Paulo (SP), Brasil.

**Keywords:** Voz, Docentes, Fatores de Risco, Disfonia, Fonoaudiologia

## Abstract

**Objetivo:**

Verificar os riscos gerais e específicos de disfonia em professores com e sem alteração vocal, comparar os escores dos protocolos de rastreio e correlacionar os riscos com o grau geral de desvio vocal (G).

**Método:**

Participaram professores de diferentes níveis de ensino, de ambos os sexos, idades acima de 18 anos. Foram aplicados o Protocolo de Risco de Disfonia - Geral (PRRD-G) e o Protocolo de Rastreio de Risco de Disfonia - Professores (PRRD-Pro). Foram gravadas e analisadas amostras vocais e os participantes foram distribuídos em dois grupos, com e sem alteração vocal, a partir da média do G no Consenso da Avaliação Perceptivo-Auditiva da Voz (CAPE-V). Realizaram-se análises descritivas e comparações dos dados entre os grupos.

**Resultados:**

Foi observada homogeneidade entre os grupos quanto ao sexo e idade. Não houve diferença estatística significativa entre os grupos nos escores finais do PRRD-G, PRRD-Pro e escore total. No entanto, observou-se diferença nos subescores de hidratação (pior no grupo não alterado) e fumo (pior no grupo alterado). Não houve correlação entre os escores dos protocolos e o G.

**Conclusão:**

Os escores médios do PRRD-G foram acima do ponto de corte nos dois grupos, indicando elevado risco de disfonia, mesmo na ausência de alteração vocal. Fumo e hidratação se mostraram relevantes na diferenciação entre os professores com e sem alteração vocal. Está prevista a continuidade do estudo com aumento do tamanho da amostra, exame laringológico, observação da voz e comunicação em sala de aula, oficinas de aprimoramento vocal e fonoterapia.

## INTRODUÇÃO

A voz na carreira docente é fundamental para a transmissão de conhecimentos aos estudantes e exige cuidados especiais. O professor, por utilizar a voz como principal instrumento de trabalho, é caracterizado como um profissional da voz falada, sendo portanto, mais suscetível ao desenvolvimento de Distúrbio de Voz Relacionado ao Trabalho-DVRT^(1)^. As características anatomofisiológicas individuais, a sobrecarga de trabalho, as condições do ambiente físico e o nível de ensino para o qual lecionam são condições que podem interferir na probabilidade de desenvolverem alterações vocais. Em razão disso, o docente pode ter que se ausentar do trabalho e sofrer impactos na sua atividade laboral e na qualidade de vida.

Dentre os sinais e sintomas vocais comuns relatados por professores nesta patologia ocupacional, estão fadiga vocal, rouquidão, sensação de incômodo no trato vocal, cansaço durante e posteriormente ao uso da voz, agravamento dos sintomas pelo uso prolongado da voz, esforço excessivo para falar, tensão e dor na região da cabeça e pescoço, diminuição da projeção vocal, episódios de afonia, quebras e falhas na voz, voz fraca, pigarro, entre outros^(2-5)^.

Todas essas manifestações podem ser identificadas e monitoradas por instrumentos de rastreio de distúrbios vocais; quanto mais presentes, maior o risco de disfonia. O Protocolo de Rastreio de Risco de Disfonia - Geral (PRRD-G), como proposta que amplia esta investigação, mostrou-se sensível para diferenciar pessoas com e sem queixa vocal ou disfonia, e permite verificar o risco de disfonia em diferentes grupos devido à sua aplicabilidade em uma ampla faixa etária, e independentemente de gênero, sexo e uso vocal. Tal protocolo engloba tópicos subjetivos e objetivos relacionados à voz, incluindo o rastreio de sinais e sintomas, histórico de doenças, hidratação, alterações de voz na família, dentre outros^(6)^.

É possível associá-lo a protocolos de rastreio de risco de disfonia específicos, de acordo com o grupo profissional, para complementar e analisar os riscos de forma mais robusta, uma vez que as profissões possuem peculiaridades quanto às demandas, necessidades e riscos no uso vocal no trabalho^(7)^. Estudo com professores do ensino infantil e fundamental, no qual foi aplicado o PRRD-G e um protocolo piloto específico de rastreio de disfonia para professores (PRRD-Pro), verificou sua sensibilidade para diferenciar as particularidades dos participantes^(6,7)^.

Tais ferramentas possibilitam rastrear, orientar, prevenir distúrbios da voz e colaboram com a prática terapêutica, além de permitirem o aprimoramento da autopercepção dos professores frente aos riscos no âmbito pessoal e laboral. Desse modo, a atenção aos subescores com maiores pontuações pode motivar mudanças de hábitos e comportamentos que promovam maior bem-estar vocal e diminuam as condições geradoras ou agravantes dos distúrbios de voz nesta população.

Apesar da voz do professor ser amplamente estudada, poucas pesquisas se propuseram a acompanhar longitudinalmente o risco de disfonia desses profissionais. Assim, esta pesquisa compreende etapas como a investigação do risco, observação em sala de aula, propostas de oficinas e fonoterapia, bem como acompanhamento longitudinal dos riscos vocais. O objetivo, nesta primeira etapa, foi investigar o risco de disfonia geral e específico em professores com e sem alteração vocal, correlacionar o risco de disfonia com o grau geral de desvio vocal e apresentar a proposta final do PRRD-Pro.

## MÉTODO

Pesquisa transversal aprovada pelo Comitê de Ética em Pesquisa da instituição, (CAAE nº 87344318.0.0000.0065). Todos os participantes assinaram o termo de consentimento livre e esclarecido.

A proposta foi apresentada a duas instituições de ensino, uma pública e uma particular, ambas no município de São Paulo, e o convite de participação feito aos professores a elas vinculados, independentemente do nível de ensino e gênero; as idades foram consideradas acima de 18 anos. Aqueles que não completaram as etapas do estudo foram excluídos.

Considerando o cálculo amostral, participaram 79 professores, divididos, de acordo com a presença de alteração vocal, em grupo controle (GC): n = 34, sendo 27 mulheres e 7 homens, com média de idade de 39,56 anos (DP = 7,64) e média do grau geral de desvio vocal (G) de 26,56 (DP = 8,53); e grupo experimental (GE): n = 45, com 35 mulheres e 10 homens, média da idade de 42,42 anos (DP = 10,44) e média do G = 45,47 (DP = 9,53). Houve distribuição semelhante entre os grupos quanto à idade e gênero, com homogeneidade para essas variáveis (p=0,157).

A coleta de dados ocorreu nas instituições de trabalho dos participantes, em ambiente controlado e silencioso, em dois momentos (estudo anterior^(7)^ e atual). No estudo anterior^(7)^ participaram professores de uma escola particular. Uma nova coleta, em uma escola pública, foi adicionada à esta amostra anterior, compondo, dessa forma, a amostra do estudo atual. Os participantes preencheram o Protocolo de Rastreio de Risco de Disfonia - Geral (PRRD-G)^(6)^ e o Protocolo de Rastreio de Risco de Disfonia - Complementar Profissional da Voz Falada - Professor (PRRD-Pro), disponibilizados de maneira impressa ou online (via *Google Forms*), com a equipe de pesquisa presente em ambos os contextos para sanar eventuais dúvidas.

Cada protocolo permite calcular subescores parciais e o escore final. Os subescores são calculados a partir da soma das pontuações atribuídas a cada item dos blocos temáticos e cada item pode variar de 0 (sem risco) a 3 (maior risco). Nas pontuações finais somam-se os subescores que podem variar de 0 a 131 (PRRD-G) e de 0 a 44 (PRRD-Pro). Os escores final do PRRD-G e do PRRD-Pro foram somados para análise do escore total.

O PRRD-G teve sua pontuação de corte para risco elevado definida no estudo de sua validação, sendo 29,25 para mulheres adultas, 22,75 para homens adultos e 27,10 para idosos de ambos os sexos^(6)^, e essas pontuações foram consideradas na presente análise.

O PRRD-Pro proposto inicialmente era constituído de 13 questões, sendo 12 voltadas para todos os professores, independentemente do sexo, e envolviam o uso diário da voz, descanso vocal, aquecimento e desaquecimento, tipo de atuação do professor (geral/regente, especialista ou coordenador), número médio de alunos por classe, nível de ensino, série escolar, tempo em que leciona, condições físicas do ambiente de trabalho, uso de recursos de amplificação vocal, afastamento do trabalho por disfonia, tabagismo, consumo de álcool, uso de drogas ilícitas, aspectos relacionados à utilização de prótese dentária e um bloco de questões específicas sobre saúde feminina.

Após o preenchimento dos protocolos foram realizadas gravações de amostras vocais. Na coleta inicial utilizou-se o gravador Zoom H4 posicionado perpendicularmente a 30 centímetros da boca do participante^(7)^. Na coleta atual foi utilizado o microfone Shure^®^ MV88+ posicionado a dez centímetros da boca^(8)^ e conectado a um Apple iPad^®^. Foram usadas as tarefas propostas no Consenso da Avaliação Perceptivo-Auditiva da Voz (CAPE-V)^(9)^ e considerado para este estudo o grau geral do desvio vocal (G).

Para este estudo a análise perceptivo-auditiva de toda a amostra foi realizada por dois fonoaudiólogos com mais de cinco anos de experiência neste tipo de análise. Foi adicionada à amostra a repetição aleatória e cega das vozes de 20 indivíduos da amostra para a análise de confiabilidade; utilizou-se o modelo de duas vias com efeitos fixos baseado na concordância absoluta de medidas únicas no cálculo do coeficiente de correlação intraclasse (CCI) e a confiabilidade interjuízes foi moderada (0,520). A partir da análise individual de cada juiz foi calculada a média simples entre ambos, gerando o valor final do grau geral do desvio vocal e extraídas média, mediana, desvio padrão, mínimo e máximo. Foi considerado sem alteração o grupo que pontuou entre 0 e 35,5 (GSA) e com alteração aqueles acima de 35,5 (GE)^(9)^.

Para análise estatística descritiva e inferencial consideraram-se as variáveis: sexo, idade, grau do desvio vocal, escore do PRRD-G, escore do PRRD-Pro e escore total (soma dos escores do PRRD-G e PRRD-Pro); subescores do PRRD-G: escala analógica visual (EAV), sinais e sintomas (SS), uso da voz fora do trabalho (VFT), alimentação (AL), hidratação (H), sono (SN), histórico de doenças (DO), alteração de voz anterior (AA), medicamentos (MD) e contato com fumantes (F); e os subescores do PRRD-Pro: tempo de atuação profissional (T), aspectos relacionados à profissão (P), condições físicas do ambiente de trabalho (CA), saúde feminina (END), fumo (F2) e prótese dentária (PD).

Para comparações entre os grupos GE e GC quanto aos escores PRRD-G, PRRD-Pro e escore total foi usado o teste t-student; para comparação dos subescores do PRRD-G e do PRRD-Pro: teste t-student*,* com fator de correção Welch e teste exato de Fisher; e para análise de correlação entre os escores dos protocolos (PRRD-G, PRRD-Pro e escore total) e o grau geral de desvio vocal (G): teste de correlação de Pearson*.*

A análise estatística adotou valor de significância de 5% (p ≤ 0,05) e utilizou-se o software SPSS Statistics, versão 27.0 (IBM Corp., Armonk, NY, EUA). Os resultados foram interpretados também com base no tamanho do efeito.

## RESULTADOS

Quanto aos escores PRRD-G, PRRD-Pro e total, observou-se proximidade das pontuações médias de cada um entre os grupos, com similaridade de risco, independentemente de presença ou não de alteração vocal. Para o escore do PRRD-G as médias ficaram acima do corte para alto risco de disfonia em ambos os grupos. Na comparação dos escores entre os grupos não houve diferença estatisticamente significativa ou tamanho de efeito relevante, conforme apresentado na Tabela 1.

**Tabela 1:** Caracterização e comparação dos grupos em relação aos escores de PRRD-G, PRRD-Pro e Escore Total

**Escores**	**GC**		**GE**		**Dif.**	**IC 95%**	**gl**	**p**	**T.E.**
	**Média (DP)**	**Mediana (Mín.–Máx.)**	**Média (DP)**	**Mediana (Mín.–Máx.)**					
PRRD-G	40,89	40,90	42,94	40,00	-2,04	-8,92–4,42	77	0,532	0,072
	(14,14)	(10–66)	(15,60)	(16–88,7)					
PRRD-Pro	21,53	22,00	21,49	22,00	0,41	-2,03–2,19	77	0,971	0,004
	(4,27)	(12–30)	(5,32)	(12–34)					
Total	62,13	66,00	64,42	61,00	-2,30	-9,22–4,96	77	0,553	0,069
	(15,96)	(34–90)	(17,95)	(31–118,70)					

**Legenda:** DP: desvio padrão; Mín.: Mínimo; Máx.: Máximo; Dif.: diferença entre as médias; IC: intervalo de confiança; gl: graus de liberdade; T.E.: tamanho do efeito

Na comparação dos grupos quanto aos subescores do PRRD-G encontrou-se diferença estatisticamente significativa na hidratação (H), ou seja, as pontuações dos professores sem alteração vocal foram maiores do que as do grupo com alteração, indicando maior risco no subescore hidratação em GSA. Na comparação dos grupos quanto aos subescores do PRRD-Pro observou-se diferença estatisticamente significativa no fumo (F), com pontuação maior dos professores com alteração vocal, indicando maior risco no subescore fumo em GE, conforme detalhado na Tabela 2.

**Tabela 2:** Caracterização e comparação dos grupos em relação aos subescores do PRRD-G e PRRD-Pro

	**GC**		**GE**		**Dif.**	**IC 95%**	**gl**	**p**	**T.E.**
	**Média (DP)**	**Mediana (Mín.–Máx.)**	**Média (DP)**	**Mediana (Mín.–Máx.)**					
**PRRD-G**									
EAV	3,69	4,00	4,69	5,00	-1,01	-2,12–0,05	77	0,073	0,209^†^
	(2,23)	(0–8)	(2,53)	(0–10)					
SS	23,35	21,50	24,73	22,00	-1,38	-6,21–3,77	77	0,611	0,017
	(11,12)	(2–46)	(11,81)	(6–59)					
VFT	2,65	3,00	2,71	3,00	-0,06	-0,58–0,48	77	0,803	0,027
	(1,28)	(0–5)	(1,06)	(0–5)					
AL	2,29	2,00	1,98	2,00	0,32	-0,21–0,82	77	0,269	0,126^†^
	(1,24)	(0–5)	(1,22)	(0–4)					
H	1,50	2,00	0,96	0,00	0,54	-0,06–1,11	77	0,047*	0,224^†^
	(1,19)	(0–3)	(1,22)	(0–3)					
SN	1,26	1,00	1,02	1,00	0,24	-0,30–0,79	63,7	0,374	0,111^†^
	(1,29)	(0–3)	(1,08)	(0–3)					
DO	1,56	1,00	1,29	1,00	0,27	-0,48–0,98	77	0,462	0,083
	(1,64)	(0–5)	(1,66)	(0–6)					
AA	1,56	2,00	1,60	2,00	-0,04	-0,35–0,30	77	0,803	0,030
	(0,70)	(0-2)	(0,72)	(0-2)					
MD	0,29	0,00	0,56	0,00	-0,26	-0,52 - 0,01	76,1	0,070	0,207^†^
	(0,58)	(0-2)	(0,69)	(0-2)					
F	0,26	0,00	0,58	0,00	-0,31	-0,67–0,04	75,2	0,088	0,199†
	(0,621)	(0-3)	(0,97)	(0-3)					
**PRRD-Pro**									
T	2,79	3,00	3,02	3,00	-0,23	-0,82–0,39	77	0,466	0,083
	(1,41)	(1–7)	(1,36)	(1–7)					
P	9,76	10,00	8,98	9,00	0,79	-0,20–1,73	77	0,116	0,176†
	(2,08)	(6–12)	(2,39)	(3–12)					
CA	6,85	7,00	7,07	7,00	-0,21	-1,33–0,82	77	0,690	0,046
	(2,11)	(3–11)	(2,80)	(2–14)					
END	0,82	1,00	0,78	1,00	0,05	-0,25–0,34	77	0,759	0,034
	(0,76)	(0–3)	(0,56)	(0–2)					
F2	0,12	0,00	0,51	0,00	-0,39	-0,78–0,00	67,2	0,041*	0,243^†^
	(0,54)	(0-3)	(1,10)	(0-3)					
PD	0,03	0,00	0,04	0,00	-0,02	-0,10–0,07	77	0,743	0,039
	(0,17)	(0-2)	(0,21)	(0-2)					

Teste t-student

* estatisticamente significativo;

† efeito pequeno

**Legenda:** DP: desvio padrão; Mín.: mínimo; Máx.: máximo; Dif.: diferença entre as médias; IC: intervalo de confiança; gl: graus de liberdade; T.E.: tamanho do efeito; EAV: escala analógica visual; SS: sinais e sintomas; VFT: voz fora do trabalho; AL: alimentação; H: hidratação; SN: sono; DO: histórico de doenças; AA: alteração anterior de voz; F: contato com fumantes; T: tempo de atuação profissional; P: características da atuação como professor; CA: condições ambientais; END: saúde feminina; F2: fumo; PD: prótese dentária

Não houve correlação estatística significativa entre a pontuação média do G e os escores do PRRD-G (p = 0,315), PRRD-Pro (p = 0,962) e total (p = 0,262), e o tamanho do efeito foi pequeno.

## DISCUSSÃO

Este estudo investigou o risco de disfonia em professores, comparou esse risco entre professores com e sem alteração vocal, além de analisar a correlação entre risco e grau geral de desvio vocal.

O grupo com alteração vocal foi composto por professores com desvios de leve a moderado, de maneira semelhante a outro trabalho com 60 professores com disfonia^(10)^. Muitas vezes o grau leve de alteração vocal pode passar despercebido a estes profissionais que acabam não procurando atendimento fonoaudiológico^(10)^.

Houve distribuição semelhante entre os grupos quanto à idade e proporção entre os sexos. Ressalta-se, no entanto, que a predominância do sexo feminino em ambos os grupos reflete a composição da população docente, e que o número reduzido de participantes do sexo masculino pode influenciar generalizações quanto à influência do sexo nos achados. Tal aspecto reforça a importância de considerar os fatores relacionados às condições de trabalho e ao uso da voz profissionalmente como primordial para análise do risco para o DVRT, considerando além das variáveis biológicas^(1,10)^.

Quanto aos dados dos protocolos, foi observado que os escores do PRRD-G e PRRD-Pro apresentaram resultados semelhantes em ambos os grupos de professores, com pontuações elevadas, apontando alto risco de disfonia. Tal achado sugere que o risco para disfonia é similar para esses profissionais, no contexto geral e específico do uso da voz, independentemente da presença de disfonia, da dinâmica de trabalho e da instituição, como observado anteriormente^(7)^. Esses dados são reforçados pela ausência de correlação entre o risco de disfonia e o grau do desvio vocal.

Por outro lado, dois subescores conseguiram diferenciar os grupos: fumo e hidratação, ainda que com efeito de tamanho pequeno.

Em relação à hidratação, os professores dos dois grupos referiram ingerir quantidades de água aquém do mínimo indicado, sendo apenas um pouco maior no grupo com alteração vocal, talvez numa tentativa de amenizar maior desconforto durante o uso da voz. Tais dados devem ser melhor explorados futuramente, e reforçada entre professores a relação entre a falta de hidratação adequada e presença de sintomas como garganta seca, pigarro e fadiga vocal^(3)^. A hidratação sistêmica, considerada ideal, é muito acessível para o professor em sala de aula^(11)^ e são conhecidos seus efeitos positivos nos aspectos vocais, inclusive quando a ingestão hídrica ocorre após carga vocal, com os participantes relatando menos esforço fonatório após se hidratarem^(12)^.

Quanto ao fumo, observou-se baixa ocorrência deste hábito em ambos os grupos, um pouco maior nos professores com alteração vocal. É consenso na literatura científica os malefícios do fumo para a saúde geral e em específico para a laringe e, consequentemente para a voz. As altas concentrações de substâncias nocivas produzem irritações, inflamações e ressecamento das mucosas, o que irá provocar mudanças nas propriedades acústicas e perceptivo-auditivas da voz^(13,14)^. Estudo com 357 docentes universitários registrou piores pontuações nos protocolos de qualidade de vida e desvantagem vocal dos indivíduos fumantes em comparação aos não fumantes, com limitações no uso da voz, inclusive durante o exercício profissional; além de aumento das queixas vocais diárias e de outros desconfortos no sistema respiratório^(14)^.

O fumo está diretamente relacionado à presença de alteração vocal, haja vista os malefícios à voz consolidados na literatura, sendo o Edema de Reinke uma das alterações laríngeas mais frequentes em tabagistas, com relevante impacto na voz^(15)^.

Ao final desta investigação preliminar do PRRD-Pro foram elencados aspectos observados pelos investigadores durante a coleta, que foram modificados na versão final (Apêndice A). Assim, foram adicionados os subitens “ensino remoto”, “saúde mental” e “aparelhos ortodônticos/alinhadores dentários”, detalhado o subitem “fumo” e modificada a pontuação dos subitens “uso de drogas” e “prótese dentária”.

Este estudo colabora com a produção científica sobre a voz do professor. Os achados reforçam a necessidade de aprofundamento na análise dos indicadores de risco de disfonia, especialmente aqueles intrínsecos à carreira docente. A associação entre os protocolos PRRD-G e PRRD-Pro, e em conjunto com a avaliação da voz, fornece dados quantitativos e qualitativos que podem nortear diretrizes de ações fonoaudiológicas futuras. Os achados referentes à hidratação e ao tabagismo evidenciam a necessidade de que esses aspectos sejam incorporados de forma prioritária em estratégias de prevenção e intervenção.

Por fim, na continuidade do estudo, além do aumento da amostra para confirmar os achados, outras formas de divisão dos grupos poderão ser exploradas, haja vista que a média de alteração vocal na presente amostra foi de grau leve. Os participantes estão sendo observados durante as aulas e também encaminhados gradativamente para exame laringológico. Todos os dados resultantes serão considerados no estudo completo.

## CONCLUSÃO

Os escores médios do PRRD-G foram acima dos pontos de corte para mulheres e homens, o que representa risco elevado de disfonia nos dois grupos de professores, com e sem alteração vocal.

Não houve diferença entre os grupos em relação aos escores médios finais (PRRD-G, PRRD-Pro e escore total) e não foi observada correlação entre o risco de disfonia e o grau geral de desvio vocal, o que demonstra que o alto risco de disfonia ocorreu independentemente da presença de alteração vocal. Somente os subescores hidratação e fumo mostraram pequenas diferenças entre os grupos.

Foi possível adequar e finalizar o protocolo de rastreio de disfonia específico para professores.

## Apêndice A Protocolo de Rastreio de Risco Disfonia – Complementar Profissional da Voz Falada – Professor

PROTOCOLO DE RASTREIO DE RISCO DISFONIA – COMPLEMENTAR

PROFISSIONAL DA VOZ FALADA – Professor

PRRD-Pro

**Instruções gerais:** as pontuações que entram no cálculo de cada subescore (T, AQ, P, CA, AFT, F, ALC, DR, PR, ONL, ML), bem como as orientações sobre essas pontuações, estão destacadas em cinza; no caso do/a professor/a atuar em mais de uma instituição, deverá ser preenchido um PRRD-Pro relativo a cada escola.

NOME: ___________________________________________ Data: _____/_____/________

1. Tempo de atuação profissional: ______________________________________________

**T:**__ 2. Tempo médio de uso da voz **por dia**:

2.1. dias úteis: trabalha __________ horas e usa a voz por __________ horas

(considere 0 = até 2 horas de uso/dia, 1 = entre 2h01 e 5hs, 2 = entre 5h01 e 8hs, 3 = mais de 8 horas de uso/dia)

2.2. finais de semana: trabalha _________ horas e usa a voz por __________ horas

(*considere 0 = até 2 horas de uso/dia, 1 = entre 2h01 e 5hs, 2 = entre 5h01 e 8hs, 3 = mais de 8 horas de uso/dia*)

2.3. faz pausas que possibilitam o descanso da voz? 1. ( ) não 0. ( ) sim; **se sim**, descreva tempo médio das pausas e frequência em que ocorrem:

**AQ:**__ 3. Prática de aquecer /desaquecer a voz:

3.1. Aquecimento ( ) não ( ) sim

3.2. Desaquecimento ( ) não ( ) sim

**Se sim** (ambos ou ao menos um dos dois), descreva (quais situações, com que frequência e quais procedimentos): _______________________________________________________

__________________________________________________________________________

considere 0 = sim, aquecimento e desaquecimento; 2 = só aquecimento ou só desaquecimento; 3 = não faz nem aquecimento nem desaquecimento)

**P:**__ 4. Descreva:

4.1. É professor/a de alguma disciplina específica (especialista) ou é professor (a) geral / regente / generalista?

( 1 )* especialista; qual disciplina: ( )** ________________________________________

( 3 )* geral/generalista/regente

( 0 )* outros; descreva: _____________________________________________________

*(*considere 0 = outros; 1 = professor especialista; 3 = professor geral/regente*)

**(*considere 1 = artes, línguas, etc.; 2 = Educação física*)

4.2. Número médio de alunos por classe / dia: ( ) ________________________________

__________________________________________________________________________

(considere 1 = Até 15 alunos; 2 = De 15 a 30 alunos; 3 = acima de 30 alunos)

4.3. Nível de ensino e série para a qual leciona atualmente: ( ) _____________________

__________________________________________________________________________

(*considere 0 = superior; 1 = Ensino médio; 2 = Ensino fundamental; 3 = Educação infantil*)

4.4. Há quanto tempo leciona para este nível de ensino/série: ( ) ____________________

__________________________________________________________________________

(*considere 0 = De 6 meses a 2 anos; 2 = De 2 a 4 anos; 3 = Mais de 4 anos*)

**CA:**__ 5. Condições ambientais no trabalho:

( 2 ) barulho (interno/externo)

( 2 ) poeira

( 2 ) ar condicionado

( 2 ) pó de giz

( 1 ) local aberto

( 1 ) local muito grande

( 1 ) ambiente muito quente

( 1 ) ambiente muito frio

( 2 ) produtos químicos irritativos

( 1 ) outro:_________________________

(*considere 0 = nenhuma indicação; 1 = se assinalados local aberto, local muito grande, ambiente muito quente, ambiente muito frio ou outros, marque 1 ponto para cada; 2 = se assinalados barulho interno/externo, poeira, ar condicionado, pós de giz ou produtos químicos irritativos, marque dois pontos para cada; para o cálculo do subescore, CA some a pontuação de todos que foram assinalados acima*)

6. Faz uso de microfone ou outro recurso de amplificação vocal?

( ) não ( ) sim; descreva (tempo de uso diário, modelo, se teve treinamento): ___________

____________________________________________________________________________________________________________________________________________________

6.1. **Se não usa**, acha necessário usar? ( ) não ( ) sim; se sim, descreva: ______________

____________________________________________________________________________________________________________________________________________________

**AFT:**__ 7. Você já se afastou do trabalho por causa da voz? ( 0 ) não ( 2 ) sim; **se sim**, descreva:

__________________________________________________________________________

**F:**__ 8. Em relação ao fumo de cigarro tradicional:

( 0 ) nunca fumou

( 0 ) ex-fumante há 10 anos ou mais; há quantos anos parou? _______________; número médio de cigarros que fumava: __________

( 1 ) ex-fumante há menos de 10 anos; há quantos anos parou? _____________; número médio de cigarros que fumava: ___________

( 3 ) fumante; há quanto tempo fuma? ______________________________________; número médio de cigarros/dia _____________

8.1. Com exceção do fumo tradicional, faz uso de outros tipos de fumo (cigarro eletrônico, charuto, cachimbo, narguilé, cigarro de palha etc.)? ( ) não ( ) sim

. cachimbo: ( 1 ) eventualmente; ( 2 ) às vezes; ( 3 ) sempre

. charuto: ( 1 ) eventualmente; ( 2 ) às vezes; ( 3 ) sempre

. cigarro de palha: ( 1 ) eventualmente; ( 2 ) às vezes; ( 3 ) sempre

. cigarrilha: ( 1 ) eventualmente; ( 2 ) às vezes; ( 3 ) sempre

. cigarro eletrônico: ( 1 ) eventualmente; ( 2 ) às vezes; ( 3 ) sempre

. narguilé: ( 1 ) eventualmente; ( 2 ) às vezes; ( 3 ) sempre

. outro: ___________ ( 1 ) eventualmente; ( 2 ) às vezes; ( 3 ) sempre

(*para o cálculo do subescore F some a pontuação de todos que foram assinalados em 8 e 8.1*)

**ALC:**__ 9. Ingere bebida alcoólica?

( 0 ) não ( 1 ) sim; se sim, descreva: tipo de bebida, quantidade e frequência de consumo:__________________________________________________________________

**DR:**__ 10. Usa ou usou drogas?

( 0 ) não ( 2 ) sim; **se sim**, descreva tipo, quantidade e frequência de uso: ______________

__________________________________________________________________________

**PR:**__ 11. Usa prótese dentária (como dentadura ou ponte; não considerar coroa e implante dentário)?

( ) não ( ) sim

**se não**: tem indicação de uso? ( ) não ( ) sim

**se sim**, descreva tipo e há quanto tempo: _________________________________________

**se sim**, tem alguma queixa em relação ao uso desta prótese? ( ) não ( ) sim; descreva: _________________________________________________________________________

11.1. Usa aparelho ortodôntico (seja fixo, móvel ou alinhadores tipo *Invisalign*)?

( ) não ( ) sim

**se não**: tem indicação de uso? ( ) não ( ) sim

**se sim**, descreva tipo e há quanto tempo: _________________________________________

**se sim**, tem alguma queixa em relação ao uso desta prótese? ( ) não ( ) sim; descreva:

__________________________________________________________________________ *(considere, tanto em 11 quanto 11.1: 0 = não usa e não tem indicação de uso; 1 = usa e tem queixa em relação ao uso; 2 = tem indicação de uso, mas não usa; para o cálculo do subescore PR some as pontuações de 11 e 11.1*)

**ONL:**__ 12. Ministra aulas online?

( ) não ( ) sim; descreva (frequência, local, disciplina, duração de média de cada aula): __________________________________________________________________________

__________________________________________________________________________

**se sim**, sente algum desconforto físico (dores nas costas, nos ombros, pescoço, etc.) durante ou após as aulas online? ( 0 ) não ( 1 ) sim; descreva: _______________________________

__________________________________________________________________________

**se sim**, sente algum desconforto vocal? ( 0 ) não ( 1 ) sim; descreva: _________________ __________________________________________________________________________

(para o cálculo do subescore ONL some as pontuações em cinza que foram assinaladas)

**ML:**__ 13. **Só para mulheres**:

13.1. apresenta sintomas de tensão pré-menstrual? ( 0 ) não ( 1 ) sim; se sim, descreva:

__________________________________________________________________________

__________________________________________________________________________

13.2. está grávida? ( 0 ) não ( 1 ) sim; se sim, tempo da gestação: _____________________

__________________________________________________________________________

13.3. encontra-se em período de menopausa ou pós-menopausa? ( 0 ) não ( 1 ) sim; se sim, há quanto tempo:________________________________________________________

__________________________________________________________________________

13.4. apresenta problemas hormonais? ? ( 0 ) não ( 1 ) sim; se sim, descreva: ____________

____________________________________________________________________________________________________________________________________________________

13.5. faz uso de anticoncepcional? ( 0 ) não ( 1 ) sim; se sim, descreva o tipo e o tempo de uso): ___________________________________________________________________

14. Gostaria de acrescentar alguma informação? ___________________________________

____________________________________________________________________________________________________________________________________________________

**ESCORE FINAL**__

**PRRD-Pro**____

_____________________
